# Mimicking the oxygen minimum zones: stimulating interaction of aerobic archaeal and anaerobic bacterial ammonia oxidizers in a laboratory-scale model system

**DOI:** 10.1111/j.1462-2920.2012.02894.x

**Published:** 2012-10-12

**Authors:** Jia Yan, Suzanne C M Haaijer, Huub J M Op den Camp, Laura Niftrik, David A Stahl, Martin Könneke, Darci Rush, Jaap S Sinninghe Damsté, Yong Y Hu, Mike S M Jetten

**Affiliations:** 1 Department of Microbiology, IWWR, Radboud University NijmegenHeyendaalseweg 135 6525, AJ Nijmegen, The Netherlands; 2 School of Environmental Science and Engineering, South China University of TechnologyGuangzhou 510006, China; 3 Department of Civil & Environmental Engineering, University of WashingtonSeattle, WA 98195-2700, USA; 4 Organic Geochemistry Group, MARUM, University of BremenLeobener Strasse D-28359, Bremen, Germany; 5 Department of Marine Organic Biogeochemistry, NIOZ Royal Netherlands Institute for Sea Research1790AB Den Burg, Texel The Netherlands

## Abstract

In marine oxygen minimum zones (OMZs), ammonia-oxidizing archaea (AOA) rather than marine ammonia-oxidizing bacteria (AOB) may provide nitrite to anaerobic ammonium-oxidizing (anammox) bacteria. Here we demonstrate the cooperation between marine anammox bacteria and nitrifiers in a laboratory-scale model system under oxygen limitation. A bioreactor containing ‘*Candidatus* Scalindua profunda’ marine anammox bacteria was supplemented with AOA (*Nitrosopumilus maritimus* strain SCM1) cells and limited amounts of oxygen. In this way a stable mixed culture of AOA, and anammox bacteria was established within 200 days while also a substantial amount of endogenous AOB were enriched. ‘*Ca*. Scalindua profunda’ and putative AOB and AOA morphologies were visualized by transmission electron microscopy and a C_18_ anammox [3]-ladderane fatty acid was highly abundant in the oxygen-limited culture. The rapid oxygen consumption by AOA and AOB ensured that anammox activity was not affected. High expression of AOA, AOB and anammox genes encoding for ammonium transport proteins was observed, likely caused by the increased competition for ammonium. The competition between AOA and AOB was found to be strongly related to the residual ammonium concentration based on *amo*A gene copy numbers. The abundance of archaeal *amo*A copy numbers increased markedly when the ammonium concentration was below 30 μM finally resulting in almost equal abundance of AOA and AOB *amo*A copy numbers. Massive parallel sequencing of mRNA and activity analyses further corroborated equal abundance of AOA and AOB. PTIO addition, inhibiting AOA activity, was employed to determine the relative contribution of AOB versus AOA to ammonium oxidation. The present study provides the first direct evidence for cooperation of archaeal ammonia oxidation with anammox bacteria by provision of nitrite and consumption of oxygen.

## Introduction

Oxygen minimum zones (OMZs), oxygen-deficient layers in oceanic water columns, constitute only 0.1% of oceanic volume (Paulmier and Ruiz-Pino, 2009) but play a crucial role (30–50%) in global oceanic nitrogen loss (Lam and Kuypers, 2011). In the conventional paradigm of the marine microbial nitrogen cycle, dinitrogen gas is converted to ammonium by nitrogen-fixing microbes, thereby supplying phytoplankton with nitrogen for biomass production. Surplus ammonium is oxidized by nitrifying microorganisms to nitrite and further to nitrate in the presence of oxygen. In the presence of sufficient electron donors, denitrifying microbes can reduce the oxidized nitrogen to dinitrogen gas in the absence of oxygen (Arrigo, 2005). For decades, these processes were regarded as the only pathways responsible for nitrogen loss in marine nitrogen cycling. However, the discovery of new processes and important players in the nitrogen cycle such as anaerobic ammonium oxidizing (anammox) bacteria and archaeal ammonia oxidizers have shown that our knowledge still needs to be extended (Jetten, 2008).

Anammox bacteria, which convert ammonium with nitrite to dinitrogen gas in the absence of oxygen, are present in significant numbers in various marine anoxic basins (Kuypers *et al*., 2003) and OMZs (Kuypers *et al*., 2005; Woebken *et al*., 2008; Galán *et al*., 2009; Lam *et al*., 2009; Stewart *et al*., 2012). The anammox process was estimated to contribute substantially (> 50%) to nitrogen loss in marine ecosystems (Kuypers *et al*., 2005; Hamersley *et al*., 1996). Different biomarkers are used to detect and quantify anammox bacteria in these ecosystems. All 16S rRNA gene sequences of anammox bacteria found in marine ecosystems affiliate with the ‘*Candidatus* Scalindua profunda’ cluster (Schmid *et al*., 2007; Woebken *et al*., 2007; van de Vossenberg *et al*., 2008; 2012). In addition to the 16S rRNA, unique ‘ladderane’ membrane lipids (Sinninghe Damsté *et al*., 2002c) represent suitable biomarkers for the detection of anammox bacteria (Jaeschke *et al*., 2007; 2009; 2010; Rattray *et al*., 2008; Pitcher *et al*., 2011; Wakeham *et al*., 2012). The source of nitrite for anammox in OMZs, characterized by very low nitrite and ammonium concentrations, has yet not been clearly identified. Recent studies indicate that nitrite can be either supplied to anammox bacteria via partial nitrate reduction (66%) or by partial nitrification (33%) (Lam *et al*., 2009). The present study focuses on partial nitrification as potential source of nitrite for ‘*Ca*. Scalindua profunda’ marine anammox bacteria.

The first and rate-limiting step of nitrification, the aerobic oxidation of ammonium to nitrite, has for a long time been assumed to be only performed by bacteria (ammonia-oxidizing bacteria, AOB) that possess the key enzyme ammonia monooxygenase (AMO). This membrane-bound enzyme is composed of three subunits (encoded by the genes *amo*A, *amo*B and *amo*C) and catalyses the initial oxidation of ammonia to hydroxylamine. The first indication for the existence of ammonia-oxidizing archaea was derived from a putative ammonia monooxygenase-encoding gene cluster associated with an archaeal scaffold detected by a metagenomic analysis on seawater (Venter *et al*., 2004). This hypothesis was confirmed by the isolation of *Nitrosopumilus maritimus*, a marine archaeon that oxidized ammonia aerobically to nitrite and contained all three subunits of the AMO (Könneke *et al*., 2005) as well as by the dominance of the *amo*A gene of AOA over that of AOB in the ocean (Wuchter *et al*., 2006). The *amo*A gene thus serves as a molecular marker to determine the diversity and abundance of AOA and AOB (e.g. Francis *et al*., 2005; Mincer *et al*., 2007; Lam *et al*., 2009). AOA may also be detected based on crenarchaeol (e.g. Sinninghe Damsté *et al*., 2002a,b; Coolen *et al*., 2007) which is a characteristic glycerol dibiphytanyl glycerol tetraether (GDGT) found in the membrane lipids of thaumarchaea or with attached polar head groups (Pitcher *et al*., 2011). Even though AOA and AOB compete for ammonium with anammox bacteria they might serve as natural partners for anammox bacteria by oxidizing just a part of ammonium to nitrite under oxygen limitation. Within the competition for ammonia between aerobic ammonia oxidizers, AOA have been shown to have an extremely high affinity (K_s_ = 133 nM) towards ammonia (Martens-Habbena *et al*., 2009), while AOB can survive oxygen deprivation for many years (van de Graaf *et al*., [Bibr b51]). However, dedicated competition experiments with cultures have not yet been performed. AOA are highly abundant in marine ecosystems (Wuchter *et al*., 2006; Mincer *et al*., 2007) and apparently can coexist with anammox bacteria in marine OMZs (Lam *et al*., 2007; Woebken *et al*., 2007; Rusch *et al*., 2009) and anoxic basins (Coolen *et al*., 2007; Wakeham *et al*., 2012) where they could provide nitrite to marine anammox bacteria. Furthermore, OMZs are characterized by low ammonium conditions (< 0.35 μM, Stewart *et al*., 2012) which may be more suitable for activity of AOA than AOB (Martens-Habbena *et al*., 2009). With their high affinities for ammonia (133 nM, Martens-Habbena *et al*., 2009) and oxygen (2–4 μM, Parker *et al*., 2010) AOA seem excellently suited to thrive in marine OMZs. In contrast, AOB appear to be more competitive under conditions of relatively high ammonium, as shown in soil mesocosms in which AOB outcompeted AOA at 200 μg ammonium g^−1^ soil (Verhamme *et al*., 2011). One aspect greatly hampering the elucidation of whether AOA or AOB may contribute more to marine nitrification is that abundance, as determined by quantification of *amo*A gene copy numbers, does not necessarily reflect an actual contribution to nitrification. Recent research (Jia and Conrad, 2009; Bernhard *et al*., 2010) found no direct correlation between AOA abundance and potential nitrification rates in both marine, estuarine and soil ecosystems whereas other studies (e.g. Beman *et al*., 2008) were able to demonstrate a correlation between AOA abundance and nitrifier activity.

As described above AOA, AOB and anammox bacteria may have both mutualistic and competing interactions under oxygen limitation, and additional research is needed to discern the cooperation and competition among these three groups. To investigate the potential interactions between aerobic and anaerobic ammonium oxidizers similar to those occurring in OMZs, an oxygen-limited marine model system was developed (Yan *et al*., 2010) and was used here to study the interaction between the marine ‘*Ca*. Scalindua profunda’ anammox bacterium (van de Vossenberg *et al*., 2008; 2012) and *N. maritimus* AOA (Könneke *et al*., 2005) under oxygen limitation. Because *Nitrosomonas*-like AOB are an indigenous component (≤ 1% of the total microbial community based on fluorescence *in situ* hybridization analysis) of ‘*Ca*. Scalindua'-dominated anammox enrichment cultures (Yan *et al*., 2010), the competition between AOA and AOB in response to ammonium concentration could also be assessed. For these purposes a *N. maritimus* pure culture and oxygen were introduced in a 2 l bioreactor containing ‘*Ca*. Scalindua profunda’ marine anammox bacteria under defined and controlled conditions. A mixed culture consisting of AOA, AOB and anammox was obtained within 200 days, and monitored by activity assays, lipids analysis, *amo*A qPCR, and transmission electron microscopy. In addition, expression levels of relevant genes were determined by massive parallel sequencing of mRNA.

## Results and discussion

### Cultivation of a mixed culture of AOA, AOB and anammox bacteria

**Fig 1:**
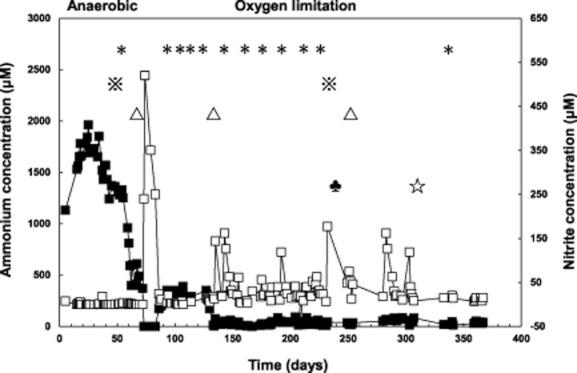
Concentrations of ammonium (▪) and nitrite (□) in the effluent of the bioreactor throughout the entire operational period. The asterisks (∗) indicate when biomass was harvested for genomic DNA isolation followed by PCR or qPCR analyses. The white triangles (▵) indicate when potential activity assays for each functional group were performed. The reference marks (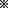) indicate when biomass was harvested for RNA isolation, and clubs (♣) and white stars (☆) indicate when biomass was harvested for TEM and lipid analyses respectively.

In order to mimick the conditions in the oxygen minimum zone as close as was technically feasible, an anaerobic preculture of ‘*Ca*. Scalindua profunda’ anammox bacteria was started and stabilized. The nitrite concentrations in the feed were increased twice, on day 34 and 59, from 10 mM to 11 mM and 12.5 mM respectively (as shown in Table 1). This resulted in a residual ammonium concentration of about 400 μM, as shown in Fig. 1, which was deemed low enough to introduce AOA cells. On day 73, the precultivated *N. maritimus* culture (Fig. S1) was transferred into the anammox bioreactor, and oxygen was introduced carefully. An initial peak in the residual oxygen concentration was observed (maximum dissolved O_2_ less than 1%) which led to a temporary accumulation of 480 μM nitrite (Fig. 1). Therefore, to ensure consumption of the residual oxygen and nitrite, the ammonium concentration of the medium was increased to 10.5 mM. This led to consumption of all residual oxygen within 2 days and resulted in a stabilized ammonium concentration in the reactor of 300 ± 30 μM. From day 130 onwards, due to the increasing activity of aerobic ammonia oxidizers, the ammonium concentration in the effluent gradually decreased to zero. On day 138 more than 500 μM nitrite and 1% oxygen accumulated (data not shown). Therefore, new medium with 11 mM ammonium was used from day 140 onwards, which resulted in depletion of oxygen within 2 days and a stable residual ammonium concentration of 30 ± 20 μM.

**Table 1:** Substrate concentrations in influent and effluent of the bioreactor

Period	Time (day)	Influent (mM)		Effluent (μM)^a^	
		Ammonium	Nitrite	Ammonium	Nitrite
Anaerobic precultivation	5–33	10	10	1712	1
34–58	10	11	1388	2
59–72	10	12.5	484	1
Oxygen-limited operation with high (∼ 300 μM) residual ammonium	73–85^b^	10	12.5	0	340
86–139	10.5	12.5	305	6
Oxygen-limited operation with low (∼ 30 μM) residual ammonium	140–370	11	12.5	35	40

a The values of ammonium and nitrite concentrations are average concentrations in the effluent during each time period.

b Period of unstable reactor operation.

### Activity of marine anammox bacteria under oxygen limitation

Freshwater anammox bacteria are known to be reversibly inhibited by low oxygen concentrations (Strous *et al*., 1997). Nevertheless, marine anammox bacteria were detected in significant amounts in OMZs, not only in the OMZ core (with O_2_ ∼ 1 μM) but also in upper layers of the OMZ where oxygen occurs in concentrations of around 20 μM (Lam *et al*., 2009; Stewart *et al*., 2012). This indicates that marine anammox bacteria may be better adapted to handle oxygen exposure than freshwater anammox bacteria investigated.

Indeed, the activity of the marine anammox bacteria in our system did not appear to suffer from exposure to oxygen. The potential anammox activity as determined in the off-line batch incubations described in the experimental procedures section was found to be stable throughout the experiment at 22 ± 2 μM NH_4_^+^ g protein^−1^ min^−1^ (as shown in Table 2) under anaerobic conditions as well as during the entire oxygen-limited operation. The abundance of the ‘*Ca*. Scalindua profunda’ anammox bacteria did not change significantly as demonstrated by the *hzs*A gene copy numbers that remained stable at around 2.6 ± 0.9 × 10^8^ copies ng DNA^−1^ as assessed by qPCR. Also the expression levels of the major catabolic anammox genes (Table 3; Fig. S2) did not change significantly when oxygen was introduced.

**Table 2:** Results of off-line potential activity analyses of the individual functional groups (μmol gprot^−1^ min^−1^)

		Anammox				AOM					
Period	Time (day)	Ammonium	Nitrite	Ammonium	Oxygen
Anaerobic precultivation	69	20.3	24.3	0	0
Oxygen-limited operation with high (∼ 300 μM) residual ammonium	134	24.1	24.4	1.3	2.6
Oxygen-limited operation with low (∼ 30 μM) residual ammonium	250	21.5	22.8	2.8	5.6

**Table 3:** Differential gene expression of anammox bacteria during the oxygen-limited period

Gene description	Gene name	Relative coverage		Ratio (oxygen limitation versus anaerobic conditions)
		Anaerobic	Oxygen limitation	
NO_3_^−^/NO_2_^−^ antiporter	*nar*K	1.7	3.7	2.1
Nitrate reductase	*nar*GH	10	10.5	1.0
NO_2_^−^ transport	*foc*A	1.2	3.3	2.8
cd1 NO_2_^−^ reductase	*nir*S	15.9	10.9	0.7
Octaheme HAO	*hao*	28.2	13.9	0.5
NH_4_^+^ transport	*amt*B	1.3	12.3	10
Hydrazine synthase	*HZS*	41.8	52.6	1.4
Octaheme HZO	*hzo*	55.5	46	0.8
Cytochrome *c* oxidase	*cbb*3	0.6	1.2	2.0
Cytochrome *c* peroxidase		0.9	3.3	3.6
Rubredoxin superoxide reductase		5.4	18.4	3.3

However, the transcriptome and lipid analysis data revealed that anammox bacteria did respond to the changed conditions in the system (Table 3). Most notably an enhanced expression of ammonium transport proteins encoded by the *amt*B genes was observed. The expression increased more than 10-fold in the oxygen-limited period when the ammonium concentration was around 30 μM. This upregulation was most likely an effect of the increasing competition for ammonium with nitrifiers. In addition, upregulated expression of several genes involved in oxidative stress (cytochrome *c* oxidase, cytochrome *c* peroxidase and rubredoxin superoxide reductase) was observed.

It has been reported that anammox bacteria contain C_18_ and C_20_ ladderane fatty acids with three or five linearly condensed cyclobutane rings (Sinninghe Damsté *et al*., 2002c; Rattray *et al*., 2008). Under anaerobic conditions, in ‘*Ca*. Scalindua'-dominated anammox enrichments, the relative abundance of C_18_[3]- and [5]-ladderane and C_20_[5] ladderane fatty acids were similar (Rattray *et al*., 2008). However, in the reactor sample representing oxygen-limited conditions the C_18_[3] ladderane fatty acid was much more dominant. This has never been observed before in reactor-cultivated anammox bacteria nor under *in situ* conditions (Table 4). Since the catabolism of anammox bacteria is assumed to occur in the anammoxosome organelle (van Niftrik *et al*., 2004), this observed composition change of ladderane lipids might help anammox bacteria to accommodate more *amt*B gene products or better adapt to oxygen exposure. GDGTs were detected (liquid chromatography mass spectrometry analysis; LC/MS), including crenarchaeol indicative for AOA (Fig. S3).

**Table 4:** Ladderane lipid contribution (%) in ‘*Candidatus* Scalindua profunda’ enrichments under anaerobic versus oxygen-limited conditions

Ladderane fatty acids	Structure	Relative contribution (%)				
		Culture		Natural environment^c^		
Anaerobic^a^	Oxygen limitation^b^	OMZ 1^d^	OMZ 2^e^	Anoxic basin^f^
C_18_[5] fatty acid	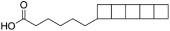	36	13	39	66	61
C_18_[3] fatty acid	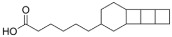	30	66	14	17	24
C_20_[5] fatty acid	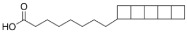	23	4	17	5	4
C_20_[3] fatty acid	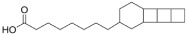	11	17	30	12	10

a Calculated from Rattray and colleagues (2008).

b Values of lipid composition under oxygen limitation were the averages of values from two replicates.

c Weighted mean of ladderane fatty acid distributions in water column particulate matter where anammox lipids have been previously reported.

d Arabian Sea Oxygen Minimum Zone, 300–750 m water depth; Rush and colleagues (2012a).

e Eastern Tropical North Pacific Oxygen Minimum Zone, four water column profiles, 55–600 m water depth; Rush and colleagues (2012b).

f Cariaco Basin, 245–346 m water depth; Wakeham and colleagues (2012).

**Fig 2:**
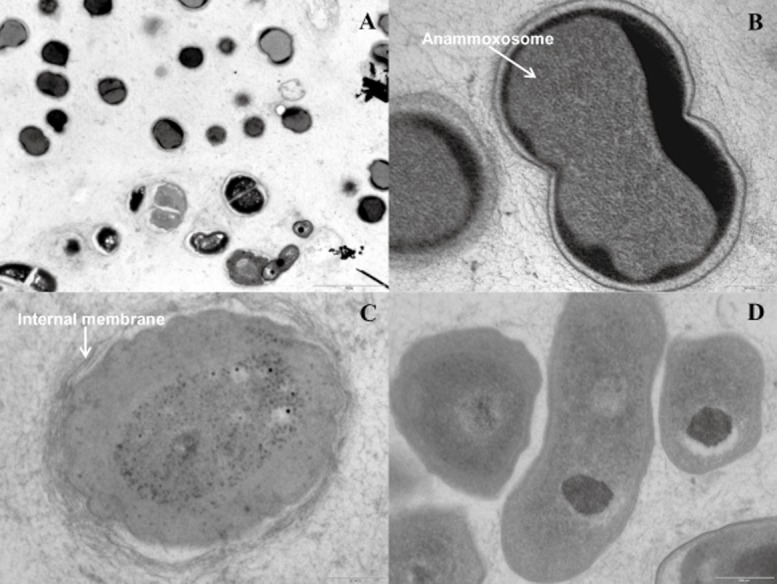
Transmission electron micrographs of the bioreactor mixed culture. A. Overview of mixed culture (AOA, AOB and anammox bacteria). B. Anammox bacterium containing the typical anammoxosome organelle. C. AOB containing the typical internal membrane structures. D. Putative AOA. The scale bars are 2 μm (A) and 200 nm (B–D).

Transmission electron microscopy was used to visualize the presence and abundance of anammox bacteria under oxygen limitation, as shown in Fig. 2A and B. As expected the majority of cells (73%) in the EM pictures were typical anammox cells showing the unique anammoxosome organelle (van Niftrik *et al*., 2008a,b).

### Abundance and competition of AOA and AOB in the mixed culture

**Fig 3:**
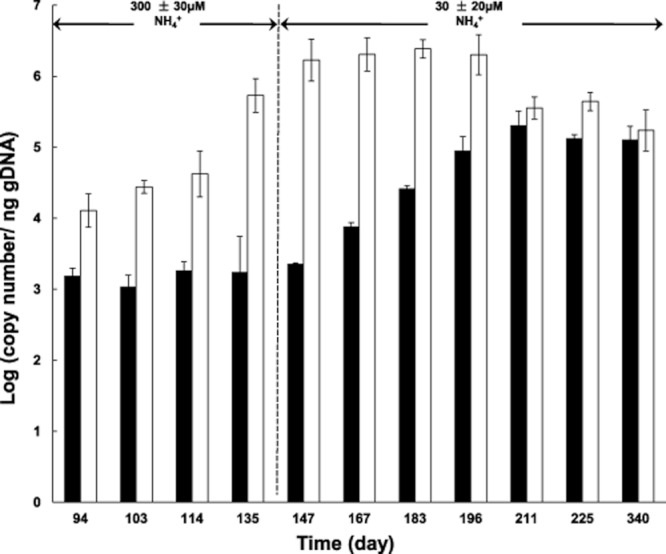
Changes on the relative abundance of *amo*A gene copy number from aerobic ammonium oxidizing-archaea (▪) and bacteria (□) in the bioreactor throughout the entire operational period in response to changing residual ammonium concentration. Values of *amo*A copy number are the averages of values from three replicate measurements.

After the introduction of *N. maritimus* cells, oxygen was introduced into the reactor system, and bacterial and archaeal *amo*A gene copy numbers were used as molecular biomarkers to monitor the growth and abundance of AOB and AOA in the mixed culture. The relative growth of AOB versus AOA based on *amo*A copy number was strongly correlated with the ammonium concentration in the bioreactor, as shown in Fig. 3. In the period of relatively high residual ammonium concentration (more than 300 μM), *amo*A gene copy numbers of indigenous AOB increased and were higher than those of the AOA. With decreasing ammonium concentration (< 30 μM), the relative as well as the absolute abundance of AOA increased (as indicated by archaeal *amo*A copy numbers). The community composition of the culture subsequently remained stable from day 211 onwards with an almost equal ratio of AOA and AOB *amo*A copy numbers of 1.6 ± 0.4 × 10^5^ and 3.2 ± 0.1 × 10^5^ copies ng DNA^−1^ respectively. In general, the introduction of oxygen into the system resulted in an increase of aerobic ammonia oxidizers. While bacterial *amo*A gene copy numbers increased 25-fold, AOA abundance increased by two orders of magnitudes since the start of oxygen addition to the culture. Changes in the population sizes suggest that the ammonium concentration controlled the competition between AOA and AOB, with low ammonium concentrations favouring the growth of AOA which is in line with the high affinity for ammonia of *N. maritimus* (K_s_ = 133 nM Martens-Habbena *et al*., 2009). In marine OMZs and other marine habitats ammonium concentrations are even more extremely limited (< 0.35 μM, Stewart *et al*., 2012) than in our bioreactor set-up which might give a further competitive advantage of AOA over AOB. The transcriptome data showed a ratio of AOB/AOA of 1.6 based on mRNA sequencing, which is in good accordance with the equal *amo*A gene copy numbers of AOA and AOB in the mixed culture. Archaeal and bacterial ammonia monooxygenase (*amo*C) and ammonium transporter (*amt*B) encoding genes were both found highly expressed under oxygen-limited conditions (as shown in Fig. S4). Interestingly, high expression of the *N. maritimus* ammonium transporter gene was also observed in the Chile OMZs, where it represented over 8% of the coding transcripts at 85 m depth (Stewart *et al*., 2012).

In the TEM (transmission electron microscope) analysis, it was possible to identify AOB cells with their typical internal membrane structures, as shown in Fig. 2A and C. Some rod-shaped, smaller microbes were found in the mixed culture, see Fig. 2A and D, which were expected to be AOA according to their size (rod-shape) and morphology (with a diameter of 0.17–0.22 mm and a length of 0.5–0.9 mm) (Könneke *et al*., 2005).

### Relative contribution of AOA and AOB to nitrification in the mixed culture

**Fig 4:**
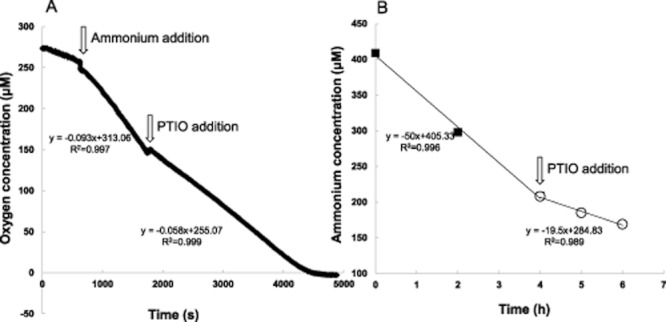
Potential aerobic ammonia-oxidizing activity of the mixed culture before (total nitrification activity) and after (bacterial nitrification activity) PTIO addition: (A) oxygen respiration and (B) ammonium consumption.

*Nitrosopumilus maritimus* was found to be inhibited by PTIO (2-phenyl-4,4,5,5-tetramethylimidazoline-1-oxyl 3-oxide; W. Martens-Habbena and D.A. Stahl, in preparation). Thus, in order to discriminate between the contribution of AOA and AOB, the inhibitor PTIO was used in batch experiments as shown in Fig. S5. Ammoniaoxidizing activity of AOA was fully inhibited, while activity of AOB was unaffected. PTIOs have been used extensively in medical research as a scavenger of free radical nitric oxide (NO) (Amano and Noda, 1995; Akaike and Maeda, 1996; Ellis *et al*., 2001). Although the mechanism of the observed differential inhibition of PTIO on the activity of AOA is not yet known, the recently proposed pathway of archaeal ammonia oxidation involving nitroxyl radicals may provide some explanations. Walker and colleagues (2010) proposed that AOA use a different ammonia oxidation pathway and, in contrast to their bacterial counterparts, do not produce hydroxylamine during the conversion of ammonia, but the reactive intermediate nitroxyl (nitroxyl hydride, HNO). This nitroxyl anion (NO^−^) could then can be rapidly oxidized to free radical NO in the presence of PTIO (Ellis *et al*., 2001). Under fully aerobic conditions AOB may not produce NO and thus their aerobic metabolism would not be inhibited by PTIO. Interestingly, Kartal and colleagues (2010) showed that also the anaerobic ammonium-oxidizing bacterium ‘*Ca*. Kuenenia stuttgartiensis’ produces NO as an intermediate and is strongly inhibited by PTIO. The different inhibition effects of PTIO on AOA and AOB, were used to estimate the contribution of each group to the total ammonium-oxidizing activity of the mixed culture. The ammonium consumption after PTIO addition was assumed to represent the contribution of bacterial nitrification only. Based on the PTIO incubations it was calculated that 40–60% of total ammonium-oxidizing activity might be attributed to AOA (Fig. 4). The lower value of 40% was obtained in the respiratory measurement, where agitation was employed, the higher value of 60% was obtained from the static incubation to determine ammonium consumption rate. This is in agreement with Martens-Habbena and colleagues (2009) who suggested that AOA are adversely affected by stirring. Because the ammonium concentration used in the assay to determine the potential aerobic ammonia oxidation is considerably higher (250 μM) than that of the reactor (< 30 μM) the actual contribution of AOA may be underestimated.

### Coupled partial nitrification–anammox from OMZs to wastewater treatment plants

Anammox bacteria and aerobic ammonia-oxidizing archaea are both recently discovered players in the nitrogen cycle (Mulder *et al*., 1995; Könneke *et al*., 2005). Their discovery has greatly changed our view on nitrogen loss from marine OMZs, where bacterial nitrification and denitrifcation were thought previously to be solely responsible for this loss. Recently, *in situ* studies indicated the presence of archaeal nitrification and anammox in various oxygen-limited ecosystems, such as the Peruvian (Lam *et al*., 2009), Chilean (Stewart *et al*., 2012), Namibia (Woebken *et al*., 2007) and Arabian Sea OMZs (Jaeschke *et al*., 2007; Jensen *et al*., 2011; Pitcher *et al*., 2011), the Black Sea (Coolen *et al*., 2007; Lam *et al*., 2007) and Cariaco Basin (Wakeham *et al*., 2012). However, because of the many interactions and contributions of different microorganisms in the nitrogen cycle, it is difficult to *in situ* target one or two microbial activities only. The mixed culture obtained in the present study, which consisted of marine AOA, AOB and anammox bacteria, provided a unprecedented opportunity to investigate the interaction of AOA, AOB and anammox bacteria under similar conditions as prevailing in OMZs. Under oxygen limsitation, low residual ammonium concentration stimulated the growth of AOA and restricted growth of AOB, and the produced nitrite was taken up by anammox bacteria and converted directly into dinitrogen gas.

Cooperation of aerobic and anaerobic ammonium-oxidizig microbes under oxygen limitation has been observed before in one-step systems (i.e. completely autotrophic nitrogen removal over nitrite, CANON) that treat ammonium-containing waste streams (Sliekers *et al*., 2002). The maximum nitrogen removal rate of 0.1 kg N m^−3^ d^−1^ obtained in the present mixed culture was relatively low compared with previous studies that used higher ammonium loading rates. Notably, low residual ammonium concentrations were obtained in the present system that might be a better reflection of the OMZ conditions. The ammonium effluent concentrations in one-step CANON systems always exceeded 0.5 mM (Sliekers *et al*., 2002; Vázquez-Padín *et al*., 2009; Bagchi *et al*., 2010; Zhang *et al*., 2010). It might be worthwhile to investigate whether cooperation between AOA and anammox occurs within, or might benefit treatment of very dilute waste streams at ambient temperature (Kartal *et al*., 2010).

## Experimental procedures

### Precultivation of *Nitrosopumilus maritimus* strain SCM1

*Nitrosopumilus maritimus* strain SCM1 (Könneke *et al*., 2005) was cultivated in a 10 l bottle with 9 l SCM medium (Könneke *et al*., 2005, 500 μM NH_4_^+^, final concentration). The incubation was performed without agitation, in the dark at room temperature (22 ± 2°C). Liquid samples (1 ml) were taken every week to monitor ammonium and nitrite concentrations in the medium. After 2 months of cultivation almost all the ammonium in the medium was converted to nitrite and the upper 8 l of the culture was pumped into another 10 l bottle (back-up culture), and the remaining concentrated cell suspension (1 l) was transferred to the marine anammox sequencing batch reactor (SBR) reactor. To check the purity of the concentrated *N. maritimus* cell suspension genomic DNA was extracted, PCR reactions targeting bacterial and archaeal *amo*A genes were performed and the resulting products were cloned and sequenced (see below). No bacterial *amo*A PCR product was obtained.

### Reactor set-up

The SBR set-up was similar to the one used in a previous study (Yan *et al*., 2010), except for the following: each SBR cycle of 24 h consisted of 22 h of filling, 1 h of settling biomass (no stirring) and 40 min of pumping off liquid from above the settled cells (0.5 l volume). During each filling period, 0.5 l of Red Sea Salt medium (van de Vossenberg *et al*., 2008) was supplemented with ammonium and nitrite (as shown in Table 1). To maintain anoxic conditions, the bioreactor and medium vessel were flushed continuously with Ar/CO_2_ (95%/5%, v/v; 20 ml min^−1^). To monitor ammonium and nitrite concentrations in the reactor, liquid samples (1 ml) were withdrawn 3–4 times every week, centrifuged (15 min at 10 000 g), and the resultant supernatant stored at −20°C until analyses.

### Operation under anaerobic conditions: precultivation of ‘*Ca*. Scalindua profunda’ marine anammox bacteria

Three hundred millilitre of ‘*Ca*. Scalindua profunda’ dominated biomass (van de Vossenberg *et al*., 2008; 2012) was transferred into the SBR set-up and incubated anaerobically (73 days) to ensure anammox activity. The influent ammonium and nitrite concentrations were adjusted until the residual ammonium concentration dropped below 500 μM (Table 1).

### Mixed-culture operation under oxygen-limited conditions

#### Operation under high residual ammonium regime

The precultivation of the marine ‘*Ca*. Scalindua profunda’ was supplemented on day 73 with the 1 l *N. maritimus* culture and the gas flow was supplemented with 1 ml min^−1^ of air. Initial nitrite accumulation indicated that the concentration of ammonium in the medium was insufficient to allow for the total consumption of supplied and produced nitrite. Therefore, the concentration of ammonium in the medium was increased whenever nitrite started to accumulate in the bioreactor until nitrite accumulation no longer occurred and a residual ammonium concentration of ∼ 400 μM was obtained (see Table 1). The reactor was operated under these conditions till day 139.

#### Operation under low residual ammonium regime

To favour the presumed high-ammonia-affinity AOA instead of low-affinity AOB, the influent ammonium and nitrite concentrations were adjusted on day 140 of SBR operation until a residual ammonium concentration of < 40 μM was obtained (see Table 1).

### Analytical methods

Protein and nitrite concentrations were quantified as described before (Yan *et al*., 2010). Ammonium concentrations were determined with the use of ortho-phtaldialdehyde (OPA) reagent (adapted from Taylor *et al*., 1974). In short, 100 μl of sample was mixed with 2 ml of diluted OPA reagent (10-fold dilution in sodium phosphate buffer, 0.3 M pH 7.3) incubated (20 min, room temperature, in the dark) and measured with a fluorescence spectrophotometer (excitation 411 nm, emission 482 nm, slit size 5 nm, 600 V). The ammonium and nitrite concentration for each liquid sample was only analysed once.

### Determinations of potential activities

Changes in potential activity for each individual functional group (anammox bacteria, aerobic ammonia and nitrite oxidizers) were determined by off-line batch incubations and respiratory measurements. Biomass from the anaerobic period (day 69) versus oxygen-limited periods (high and low residual ammonium regime; days 134 and 250) was used as indicated by the white triangles in Fig. 1. Potential activities of anammox bacteria, aerobic ammonia- and nitrite-oxidizers were determined as previously described (Yan *et al*., 2010), except for the following: final concentrations of respectively 200 μM and 500 μM ammonium and nitrite were used to test the aerobic ammonia- and nitrite-oxidizing activities in both off-line batch incubations and oxygen respiration measurements.

### Incubation in the presence of PTIO

To evaluate the relative contribution of AOA and AOB to nitrification, on day 340 (oxygen-limited operation under low ammonium regime) the potential aerobic ammonia-oxidizing activity was investigated, in the presence of PTIO (2-phenyl-4,4,5,5-tetramethylimidazoline-1-oxide-3-oxyl; MP biomedical, France) to distinguish between bacterial and archaeal activity. *N. maritimus* back-up culture (see precultivation of *N. maritimus*, strain SCM1) and indigenous *Nitrosomonas*-like AOB enrichments were used as controls to investigate the effect of PTIO, a NO scavenger (Amano and Noda, 1995; Akaike and Maeda, 1996; Ellis *et al*., 2001), on the activity of AOA and AOB. The indigenous *Nitrosomonas*-like AOB was enriched from the reactor of a preliminary experiment described previously (Yan *et al*., 2010) as follows: 4.5 ml co-culture was used as the inoculum for an 80 ml incubation. The medium contained Red Sea Salt as previously described (Yan *et al*., 2010). Incubation was performed without agitation, in the dark at room temperature (22 ± 2°C). This incubation was maintained in the lab, and every week 40 ml medium was replenished to the enrichments with fresh medium contains 250 μM ammonium after settling of the biomass. Inhibition analyses of mixed culture nitrifiers were performed in both off-line batch incubations without agitation (ammonium consumption) and through respiratory measurements (oxygen consumption). To evaluate the relative nitrification contribution of AOA and AOB, substrate consumption rates were detected before and after PTIO addition (200 μM). To calculate the relative contribution of AOA, the rate prior to PTIO addition was regarded to be 100% (representing AOB and AOA activity) and the residual rate after PTIO addition (representing AOB activity only) was subtracted to yield the percentile contribution of the AOA.

### DNA extraction, (q)PCR and sequencing analyses

The aerobic ammonia oxidizer community composition of the bioreactor was analysed through *amo*A gene end-point PCR followed by cloning and sequencing. The competition between aerobic AOB and archaea was monitored by qPCR targeting *amo*A genes. Abundance of anammox bacteria was assessed by hydrazine synthase (HZS) gene-based q-PCR. For these purposes high molecular weight DNA was extracted from biomass from the anaerobic operation (day 63), oxygen-limited operation under the high ammonium regime (days 94, 103, 114, 135) and the oxygen-limited operation under the low ammonium regime (days 147, 167, 183, 196, 225 and 340) as indicated by asterisks in Fig. 1. High molecular weight DNA was extracted from 2 ml bioreactor biomass according to the protocol described in Yan and colleagues (2010). The isolated total DNA was loaded on an agarose gel to check the quality, and analysed on a NanoDrop ND-1000 spectrophotometer (Lifescience, USA) to determine the concentration. For all samples, 2 ml of reactor content was used for DNA extraction. The concentration of isolated DNA was always 70 ± 10 μg ml^−1^. End-point PCR, cloning, sequencing and phylogenetic analyses were performed as described previously (see Table S1 for primer details). Real-time quantitative PCR was performed with an iCycler iQ5 thermocycler equipped with a real-time detection system (Bio-Rad, CA, USA). Each PCR mixture (25 μl) consisted of 12.5 μl of 2× SYBR Green PCR master mix (Finnzymes, Finland), 1 μl of forward and reverse primers (20 pmol ml^–1^) and 1 μl of template DNA (2–10 ng) per well. PCR amplification and quantification were performed in MicroAmp Optical 96-well reaction plates (Bio-Rad, CA, USA). Thermocycling for crenarchaeal *amo*A gene qPCR detection (128 bp product) was performed as follows: initial denaturation 95°C for 3 min; amplification for 40 cycles consisting of, denaturation at 95°C for 1 min, primer annealing at 58°C for 1 min, extension at 72°C for 1 min, and followed by a final elongation at 72°C for 5 min. Melting curve analysis showed only one peak at Tm = 80°C. For detection of bacterial *amo*A genes (490 bp) thermocycling consisting of: initial denaturation 96°C for 3 min; amplification for 40 cycles consisting of, denaturation at 96°C for 1 min, primer annealing at 57.5°C for 1 min, extension at 72°C for 1 min, and followed by a final elongation at 72°C for 5 min. Melting curve analysis showed only one peak at Tm = 81°C. A primer set targeting the hydrazine synthase gene (*hzs*A) was used to quantify anammox bacteria, resulting in a 226 bp product. Thermocycling was performed as follows: initial denaturation 95°C for 3 min; amplification for 40 cycles consisting of, denaturation at 95°C for 1 min, primer annealing at 55°C for 1 min, extension at 72°C for 1 min, followed by a final elongation at 72°C for 5 min. Only one peak was detected in the melting curve analysis at Tm = 80°C. No detectable peaks that were associated with primer–dimer artefacts or non-specific PCR amplification products were observed. Quantification standard curves were constructed from series of 10-fold dilutions of sequenced plasmids (DNA copy numbers ranging from 10^8^ to 10^3^ per reaction), with insert of the *amo*A genes of *N. maritimus* and *Nitrosomonas*-like AOB and the *hzs*A gene of *‘Ca*. Scalindua profunda’ anammox bacteria respectively. The amplification efficiencies were between 95% and 105%, with R^2^ value ranging from 0.994 to 0.999.

### Transcriptomics

The expression of relevant genes was determined in samples from days 48 (anaerobic condition) and 232 (the oxygen-limited operation under the low ammonium regime), by extraction of total RNA, reverse transcription and sequencing of cDNA by Illumina technology (indicated by the reference mark in Fig. 1) as described by van de Vossenberg and colleagues (2012). Total RNA isolation was performed using the RiboPure™-Bacteria kit (Ambion, Austin, USA) according to the supplier's instructions (DNase treatment was performed twice). The isolated total RNA was loaded on gel to check the quality, and analysed on a NanoDrop 1000 spectrophotometer to determine the concentration. Reverse transcription was performed using the RevertAidTM First Strand cDNA Synthesis kit (Fermentas GMBH, St Leon-Rot, Germany) with random hexamer primers according to the supplier's instructions. Second strand cDNA synthesis was performed following the suppliers instructions. At least 20 ng double-stranded cDNA was sent for Illumina sequencing to the Department of Molecular Biology of the Radboud University Medical Center Nijmegen. All reads identified as rRNA were removed prior to further analysis. The 72 nt reads were mapped onto the genomes of *N. maritimus* SCM1 (NC_010085.1), *Nitrosomonas eutropha* C91 (NC_008344.1) and ‘*Ca*. Scalindua profunda’ (van de Vossenberg *et al*., 2012) using the CLC Genomics Workbench software and the gene expression of AOA, AOB and anammox bacteria were analysed (Fig. S2; van de Vossenberg *et al*., 2012). The coverage of each gene was calculated by each gene read times 72 (read length) and divided by the length of the gene. To compare the expression under anaerobic and oxygen-limited conditions, relative gene coverage was used, which was obtained by dividing the target gene coverage with the average coverage of all genes. The gene expression ratio is the ratio of relative coverage of each gene under anaerobic condition and oxygen-limited condition.

### Lipid extraction and identification

On day 230 (oxygen-limited operation under the low ammonium regime), 20 ml of concentrated biomass was harvested from the reactor by centrifugation (10 min at 10 000 g, 4°C) and freeze-dried for lipid analyses (indicated by a white star in Fig. 1). The freeze-dried mixed culture was extracted using a modified Bligh–Dyer method (Bligh and Dyer, 1959). The sample was ultrasonically extracted for 15 min using a volume ratio of 2:1:0.8 (v/v) [methanol (MeOH) : dichloromethane (DCM) : phosphate buffer, pH 7.4]. The supernatant was collected and the residue was re-extracted ultrasonically twice. The solvent ration of the combined supernatants was adjusted to 1:1:0.9 (v/v) (MeOH : DCM : phosphate buffer) and centrifuged. The bottom DCM layer was collected and the remaining solvent re-extracted twice with DCM. The DCM layers were combined and dried to near-dryness under rotary evaporator.

Ladderane lipids analysis was performed by saponification of an aliquot of the Bligh–Dyer extract by refluxing with aqueous KOH (in 96% MeOH) at 100°C for 1 h. Fatty acids were obtained by acidifying the solution to pH 3 with 1 M HCl in MeOH and extracted using DCM. The fatty acids were converted to their corresponding fatty acid methyl esters (FAMEs) by methylation with diazomethane (CH_2_N_2_). Excess CH_2_N_2_ was removed by evaporation under N_2_. Polyunsaturated fatty acids were removed by eluting the sample over a small AgNO_3_ (5%) impregnated silica column with DCM. The fatty acid fraction was dissolved in acetone, filtered through a 0.45 μm, 4 mm diameter PTFE filter and analysed by HPLC/APCI-MS/MS (high-performance liquid chromatography coupled to positive ion atmospheric pressure chemical ionization tandem mass spectrometry) in SRM (selective reaction monitoring) mode as described in Hopmans and colleagues (2006) and modified in Rattray and colleagues (2008).

For GDGTs lipids analysis, the Bligh–Dyer extract was hydrolysed by refluxing with 2 M HCl/MeOH (1/1, v/v) for 3 h. The pH of the solution was adjusted to pH 5 using 1 M KOH (in 96% MeOH). GDGT core lipids were extracted three times using DCM. The extract was eluted over Na_2_SO_4_ dried under N_2_. A known amount of internal standard (C46 GDGT) was added to the sample before it was filtered through a 0.45 μm, 4 mm diameter PTFE filter using hexane : isopropanol (99:1). GDGTs were analysed and quantified by HPLC/APCI-MS in single ion mode (Schouten *et al*., 2007).

### Transmission electron microscopy

To visualize different morphologies of the co-culture, on day 310 (oxygen-limited operation under the low ammonium regime), 40 ml reactor sample was taken and cryofixed by high pressure freezing, freeze-substituted in acetone containing 2% osmium tetroxide and acetone containing 2% osmium tetroxide, 0.2% uranyl acetate and 1% water, embedded in Epon resin and sectioned using an ultramicrotome for TEM analysis (indicated by clubs in Fig. 1). Sample preparation was performed as previously described by van Niftrik and colleagues (2008b).

## Supporting Information

**Fig S1**Concentrations of ammonium (•) and nitrite (□) in the preculture of *Nitrosopumilus maritimus*. **Fig S2**Gene expression of 'Ca. Scalindua profunda' ananmmox bacteria under anoxic versus oxic condition. RPKM = number of reads per kb of transcript per million mapped reads. **Fig S3**Base peak chromatogram of LC/MS analysis of GDGTs in the bioreactor. Concentrations of specific GDGTs and their structures are indicated. **Fig S4**Gene expression of selected Nitrosopumilus maritimus SCM1 genes under oxygen limitation. **Fig S5**Potential aerobic ammonia-oxidizing activity of the Nitrosopumilus maritimus culture (A) and Nitrosomonas-like AOB enrichment (B). Open squares (□) and closed squares (▪) indicate AOB enrichment with and without PTIO addition respectively.

Table S1 Primers for PCR amplification and sequencing.

## References

[b1] Akaike T, Maeda H (1996). Quantitation of nitric oxide using 2-phenyl-4,4,5,5-tetramethylimidazoline-1-oxyl 3-oxide (PTIO). Methods Enzymol.

[b2] Amano F, Noda T (1995). Improved detection of nitric-oxide radical (NO-center-dot) production in an activated macrophage culture with a radical scavenger, carboxy PTIO, and Griess reagent. FEBS Lett.

[b3] Arrigo KR (2005). Marine microorganisms and global nutrient cycles. Nature.

[b4] Bagchi S, Biswas R, Nandy T (2010). Alkalinity and dissolved oxygen as controlling parameters for ammonia removal through partial nitritation and ANAMMOX in a single-stage bioreactor. J Ind Microbiol Biotechnol.

[b5] Beman JM, Popp BN, Francis CA (2008). Molecular and biogeochemical evidence for ammonia oxidation by marine Crenarchaeota in the Gulf of California. ISME J.

[b66] Bernhard AE, Landry ZC, Blevins A, de la Torre JR, Giblin AE, Stahl DA (2010). Abundance of ammonia-oxidizing archaea and bacteria along an estuarine salinity gradient in relation to potential nitrification rates. Appl Environ Microbiol.

[b6] Bligh EG, Dyer WJ (1959). A rapid method of total lipid extraction and purification. Can J Biochem Physiol.

[b7] Coolen MJL, Abbas B, van Bleijswijk J, Hopmans EC, Kuypers MMM, Wakeham SG (2007). Putative ammonia-oxidizing Crenarchaeota in suboxic waters of the Black Sea: a basin-wide ecological study using 16S ribosomal and functional genes and membrane lipids. Environ Microbiol.

[b8] Ellis A, Lu H, Li CG, Rand MJ (2001). Effects of agents that inactivate free radical NO (NO center dot) on nitroxyl anion-mediated relaxations, and on the detection of NO center dot released from the nitroxyl anion donor Angeli's salt. Br J Pharmacol.

[b9] Francis CA, Roberts KJ, Beman JM, Santoro AE, Oakley BB (2005). Ubiquity and diversity of ammonia-oxidizing archaea in water columns and sediments of the ocean. Proc Natl Acad Sci USA.

[b10] Galán A, Molina V, Thamdrup B, Woebken D, Lavik G, Kuypers MMM (2009). Anammox bacteria and the anaerobic oxidation of ammonium in the oxygen minimum zone off northern Chile. Deep Sea Res Part II Top Stud Oceanogr.

[b51] van de Graaf AA, de Bruijn P, Robertson LA, Jetten MSM, Kuenen JG (1996). Autotrophic growth of anaerobic ammonium-oxidizing micro-organisms in a fluidized bed reactor. Microbiology.

[b12] Hamersley MR, Lavik G, Woebken D, Rattray JE, Lam P, Hopmans EC (2007). Anaerobic ammonium oxidation in the Peruvian oxygen minimum zone. Limnol Oceanogr.

[b13] Hopmans EC, Kienhuis MVM, Rattray JE, Jaeschke A, Schouten S, Sinninghe Damsté JS (2006). Improved analysis of ladderane lipids in biomass and sediments using high-performance liquid chromatography/atmospheric pressure chemical ionization tandem mass spectrometry. Rapid Commun Mass Spectrom.

[b15] Jaeschke A, Hopmans EC, Wakeham SG, Schouten S, Sinninghe Damsté JS (2007). The presence of ladderane lipids in the oxygen minimum zone of the Arabian Sea indicates nitrogen loss through anammox. Limnol Oceanogr.

[b16] Jaeschke A, Op den Camp HJM, Harhangi H, Klimiuk A, Hopmans EC, Jetten MSM (2009). 16S rRNA gene and lipid biomarker evidence for anaerobic ammonium-oxidizing bacteria (anammox) in California and Nevada hot springs. FEMS Microbiol Ecol.

[b14] Jaeschke A, Abbas B, Zabel M, Hopmans EC, Schouten S, Sinninghe Damsté JS (2010). Molecular evidence for anaerobic ammonium-oxidizing (anammox) bacteria in continental shelf and slope sediments off northwest Africa. Limnol Oceanogr.

[b17] Jensen MM, Lam P, Revsbech NP, Nagel B, Gaye B, Jetten MSM, Kuypers MMM (2011). Intensive nitrogen loss over the Omani Shelf due to anammox coupled with dissimilatory nitrite reduction to ammonium. ISME J.

[b18] Jetten MSM (2008). The microbial nitrogen cycle. Environ Microbiol.

[b19] Jia ZJ, Conrad R (2009). Bacteria rather than Archaea dominate microbial ammonia oxidation in an agricultural soil. Environ Microbiol.

[b20] Kartal B, Kuenen JG, van Loosdrecht MCM (2010). Sewage treatment with anammox. Science.

[b21] Könneke M, Bernhard AE, Torre JR, Walker CB, Waterbury JB, Stahl DA (2005). Isolation of an autotrophic ammonia-oxidizing marine archaeon. Nature.

[b23] Kuypers MMM, Sliekers AO, Lavik G, Schmid M, Jorgensen BB, Kuenen JG (2003). Anaerobic ammonium oxidation by anammox bacteria in the Black Sea. Nature.

[b22] Kuypers MMM, Lavik G, Woebken D, Schmid M, Fuchs BM, Amann R (2005). Massive nitrogen loss from the Benguela upwelling system through anaerobic ammonium oxidation. Proc Natl Acad Sci USA.

[b25] Lam P, Kuypers MMM (2011). Microbial nitrogen cycling processes in oxygen minimum zones. Ann Rev Mar Sci.

[b24] Lam P, Jensen MM, Lavik G, McGinnis DF, Muller B, Schubert CJ (2007). Linking crenarchaeal and bacterial nitrification to anammox in the Black Sea. Proc Natl Acad Sci USA.

[b26] Lam P, Lavik G, Jensen MM, van de Vossenberg J, Schmid M, Woebken D (2009). Revising the nitrogen cycle in the Peruvian oxygen minimum zone. Proc Natl Acad Sci USA.

[b28] Martens-Habbena W, Berube PM, Urakawa H, de la Torre HJR, Stahl DA (2009). Ammonia oxidation kinetics determine niche separation of nitrifying Archaea and Bacteria. Nature.

[b29] Mincer TJ, Church MJ, Taylor LT, Preston C, Kar DM, DeLong EF (2007). Quantitative distribution of presumptive archaeal and bacterial nitrifiers in Monterey Bay and the North Pacific Subtropical Gyre. Environ Microbiol.

[b31] Mulder A, van de Graaf AA, Robertson LA, Kuenen JG (1995). Anaerobic ammonium oxidation discovered in denitrifying fluidized-bed reactor. FEMS Microbiol Ecol.

[b9001] van Niftrik LA, Fuerst JA, Damste JSS, Kuenen JG, Jetten MSM, Strous M (2004). The anammoxosome: an intracytoplasmic compartment in anammox bacteria. FEMS Microbiol Lett.

[b54] van Niftrik L, Geerts WJC, van Donselaar EG, Humbel BM, Webb RI, Fuerst JA (2008a). Linking ultrastructure and function in four genera of anaerobic ammonium-oxidizing bacteria: cell plan, glycogen storage, and localization of cytochrome c proteins. J Bacteriol.

[b55] van Niftrik L, Geerts WJC, van Donselaar EG, Humbel BM, Yakushevska A, Verkleij AJ (2008b). Combined structural and chemical analysis of the anammoxosome: a membrane-bounded intracytoplasmic compartment in anammox bacteria. J Struct Biol.

[b32] Parker CT, Huynh S, Quinones B, Harris LJ, Mandrell RE (2010). Comparison of genotypes of *Salmonella enterica* serovar enteritidis phage type 30 and 9c strains isolated during three outbreaks associated with raw almonds. Appl Environ Microbiol.

[b33] Paulmier A, Ruiz-Pino D (2009). Oxygen minimum zones (OMZs) in the modern ocean. Prog Oceanogr.

[b34] Pitcher A, Villanueva L, Hopmans EC, Schouten S, Reichart GJ, Sinninghe Damsté JS (2011). Niche segregation of ammonia-oxidizing archaea and anammox bacteria in the Arabian Sea oxygen minimum zone. ISME J.

[b35] Rattray JE, van de Vossenberg J, Hopmans EC, Kartal B, van Niftrik L, Rijpstra WIC (2008). Ladderane lipid distribution in four genera of anammox bacteria. Arch Microbiol.

[b37] Rusch A, Hannides AK, Gaidos E (2009). Diverse communities of active Bacteria and Archaea along oxygen gradients in coral reef sediments. Coral Reefs.

[b38] Rush D, Hopmans EC, Wakeham SG, Schouten S, Sinninghe Damsté JS (2012a). Occurrence and distribution of ladderane oxidation products in different oceanic regimes. Biogeosciences.

[b40] Rush D, Wakeham SG, Hopmans EC, Schouten S, Sinninghe Damsté JS Biomarker evidence for anammox in the oxygen minimum zone of the Eastern Tropical North Pacific. Org Geochem.

[b42] Schmid MC, Risgaard-Petersen N, van de Vossenberg J, Kuypers MMM, Lavik G, Petersen J (2012b). Anaerobic ammonium-oxidizing bacteria in marine environments: widespread occurrence but low diversity. Environ Microbiol.

[b43] Schouten S, van der Meer MTJ, Hopmans EC, Rijpstra WIC, Reysenbach AL, Ward DM (2007). Archaeal and bacterial glycerol dialkyl glycerol tetraether lipids in hot springs of Yellowstone National Park. Appl Environ Microbiol.

[b44] Sinninghe Damsté JS, Rijpstra WIC, Hopmans EC, Prahl FG, Wakeham SG, Schouten S (2002a). Distribution of membrane lipids of planktonic Crenarchaeota in the Arabian Sea. Appl Environ Microbiol.

[b45] Sinninghe Damsté JS, Schouten S, Hopmans EC, van Duin ACT, Geenevasen JAJ (2002b). Crenarchaeol: the characteristic core glycerol dibiphytanyl glycerol tetraether membrane lipid of cosmopolitan pelagic crenarchaeota. J Lipid Res.

[b46] Sinninghe Damsté JS, Strous M, Rijpstra WIC, Hopmans EC, Geenevasen JAJ, van Duin ACT (2002c). Linearly concatenated cyclobutane lipids form a dense bacterial membrane. Nature.

[b47] Sliekers AO, Derwort N, Gomez JLC, Strous M, Kuenen JG, Jetten MSM (2002). Completely autotrophic nitrogen removal over nitrite in one single reactor. Water Res.

[b48] Stewart FJ, Ulloa O, DeLong EF (2012). Microbial metatranscriptomics in a permanent marine oxygen minimum zone. Environ Microbiol.

[b49] Strous M, van Gerven E, Kuenen JG, Jetten M (1997). Effects of aerobic and microaerobic conditions on anaerobic ammonium-oxidizing (Anammox) sludge. Appl Environ Microbiol.

[b50] Taylor S, Ninjoor V, Dowd DM, Tappel AL (1974). Cathepsin B2 measurement by sensitive fluorometric ammonia analysis. Anal Biochem.

[b56] Vázquez-Padín JR, Pozo MJ, Jarpa M, Figueroa M, Franco A, Mosquera-Corral A (2009). Treatment of anaerobic sludge digester effluents by the CANON process in an air pulsing SBR. J Hazard Mater.

[b57] Venter JC, Remington K, Heidelberg JF, Halpern AL, Rusch D, Eisen JA (2004). Environmental genome shotgun sequencing of the Sargasso Sea. Science.

[b58] Verhamme DT, Prosser JI, Nicol GW (2011). Ammonia concentration determines differential growth of ammonia-oxidising archaea and bacteria in soil microcosms. ISME J.

[b52] van de Vossenberg J, Rattray JE, Geerts W, Kartal B, van Niftrik L, van Donselaar EG (2008). Enrichment and characterization of marine anammox bacteria associated with global nitrogen gas production. Environ Microbiol.

[b53] van de Vossenberg J, Woebken D, Maalcke WJ, Wessels HJCT, Dutilh BE, Kartal B (2012). The metagenome of the marine anammox bacterium ‘*Candidatus* Scalindua profunda’ illustrates the versatility of this globally important nitrogen cycle bacterium. Environ Microbiol.

[b59] Wakeham SG, Turich C, Schubotz F, Podlaska A, Li XN, Varela R (2012). Biomarkers, chemistry and microbiology show chemoautotrophy in a multilayer chemocline in the Cariaco Basin. Deep Sea Res Part I Oceanogr Res Pap.

[b60] Walker CB, de la Torre JR, Klotz MG, Urakawa H, Pinel N, Arp DJ (2010). *Nitrosopumilus maritimus* genome reveals unique mechanisms for nitrification and autotrophy in globally distributed marine crenarchaea. Proc Natl Acad Sci USA.

[b61] Woebken D, Fuchs BA, Kuypers MAA, Amann R (2007). Potential interactions of particle-associated anammox bacteria with bacterial and archaeal partners in the Namibian upwelling system. Appl Environ Microbiol.

[b62] Woebken D, Lam P, Kuypers MMM, Naqvi SWA, Kartal B, Strous M (2008). A microdiversity study of anammox bacteria reveals a novel *Candidatus* Scalindua phylotype in marine oxygen minimum zones. Environ Microbiol.

[b63] Wuchter C, Abbas B, Coolen MJL, Herfort L, van Bleijswijk J, Timmers P (2006). Archaeal nitrification in the ocean. Proc Natl Acad Sci USA.

[b64] Yan J, Op den Camp HJM, Jetten MSM, Hu YY, Haaijer SCM (2010). Induced cooperation between marine nitrifiers and anaerobic ammonium-oxidizing bacteria by incremental exposure to oxygen. Syst Appl Microbiol.

[b65] Zhang DJ, Cai Q, Cong LY (2010). Enhancing completely autotrophic nitrogen removal over nitrite by trace NO_2_ addition to an AUSB reactor. J Chem Technol Biotechnol.

