# Supplementary material for: Mimicking the oxygen minimum zones: stimulating interaction of aerobic archaeal and anaerobic bacterial ammonia oxidizers in a laboratory-scale model system
Source: Environ Microbiol. 2012 Oct 12;14(12):3146–58. doi: 10.1111/j.1462-2920.2012.02894.x (PMC3558802; doi:10.1111/j.1462-2920.2012.02894.x)
Supplement: Supplementary file 1 — Fig S1Concentrations of ammonium (•) and nitrite (□) in the preculture of Nitrosopumilus maritimus. Fig S2Gene expression of 'Ca. Scalindua profunda' ananmmox bacteria under anoxic versus oxic condition. RPKM = number of reads per kb of transcript per million mapped reads. Fig S3Base peak chromatogram of LC/MS analysis of GDGTs in the bioreactor. Concentrations of specific GDGTs and their structures are indicated. Fig S4Gene expression of selected Nitrosopumilus maritimus SCM1 genes under oxygen limitation. Fig S5Potential aerobic ammonia-oxidizing activity of the Nitrosopumilus maritimus culture (A) and Nitrosomonas-like AOB enrichment (B). Open squares (□) and closed squares (▪) indicate AOB enrichment with and without PTIO addition respectively. [file emi0014-3146-SD1.pdf]

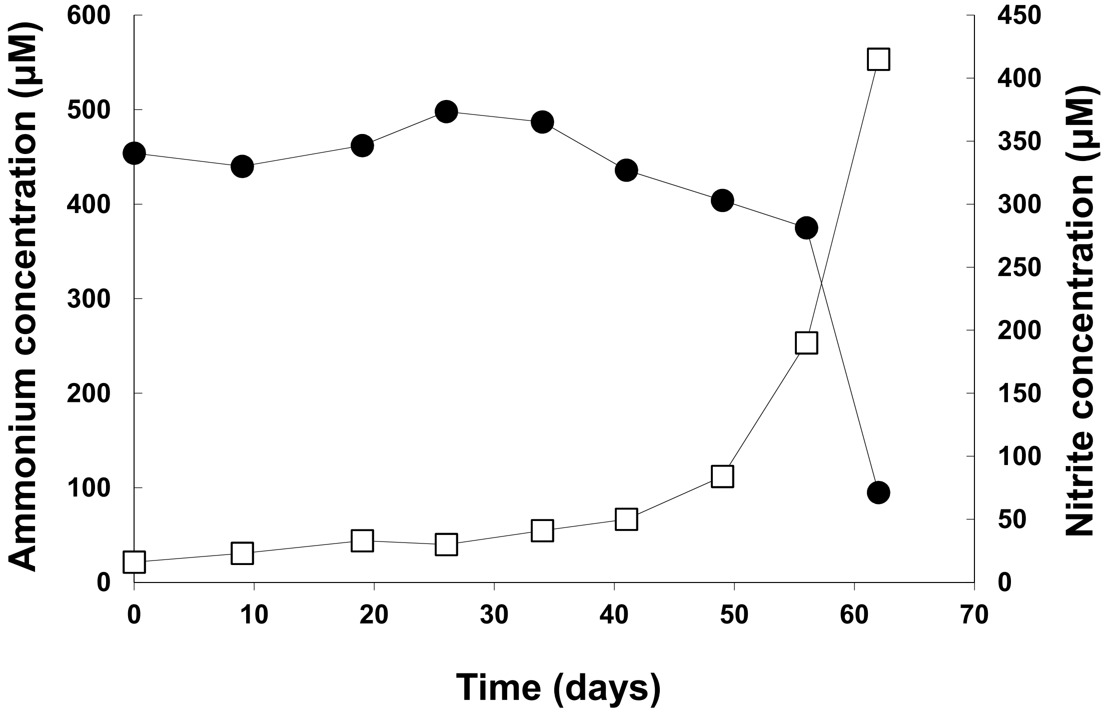

Fig. S1. Concentrations of ammonium (●) and nitrite (□) in the preculture of *Nitrosopumilus maritimus*.

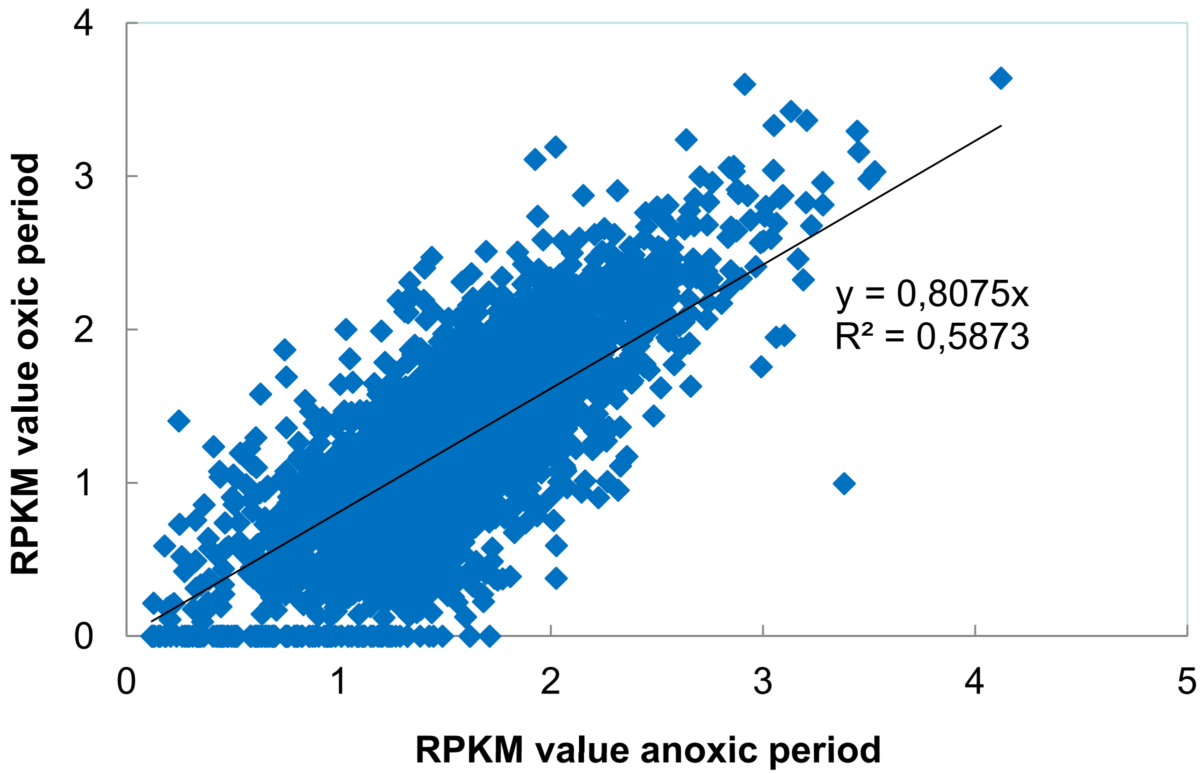

Fig. S2. Gene expression of "*Ca.* Scalindua profunda" ananmmox bacteria under anoxic versus oxic condition. RPKM = number of reads per kb of transcript per million mapped reads.

m/z	GDGT	Structure	concentration (µg·gdry weight biomass^{-1})
1302	0	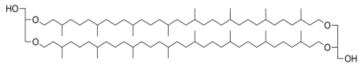	0.81
1300	1	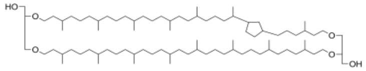	0.38
1298	2	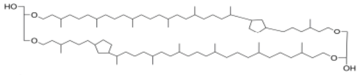	0.77
1296	3	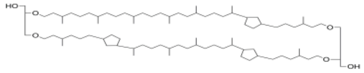	0.27
1292	crenarchaeol	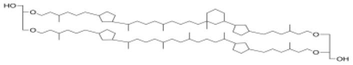	2.56

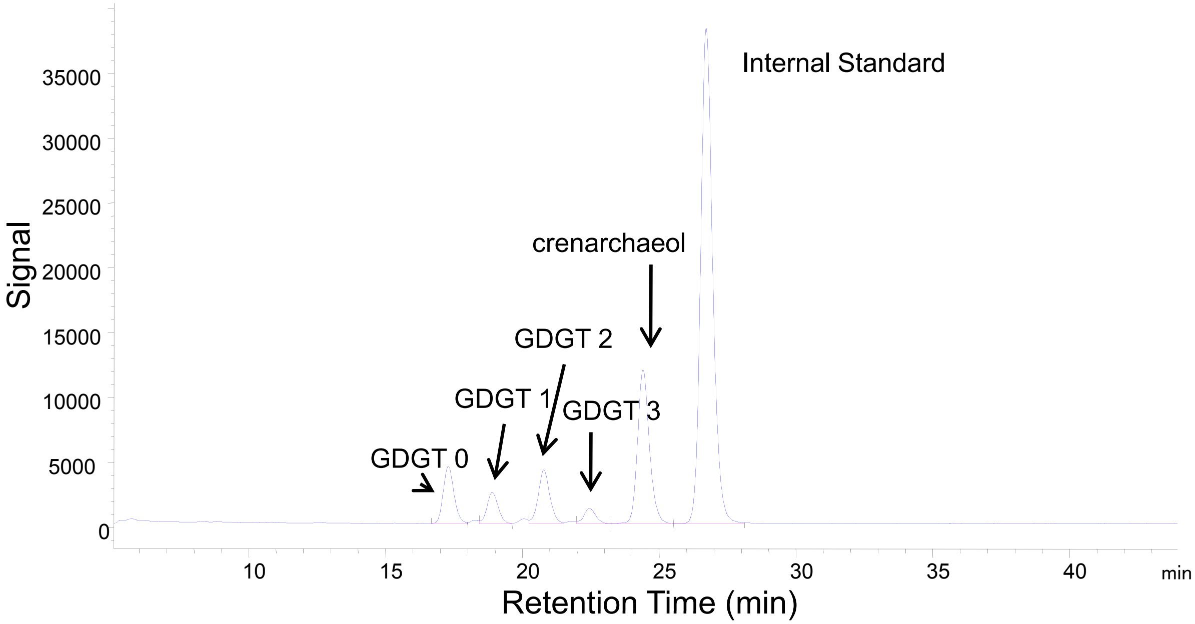

Fig. S3. Base peak chromatogram of LC/MS analysis of GDGTs in the bioreactor. Concentrations of specific GDGTs and their structures are indicated.

Description	Gene	ID	Reads
NH_4^+ transport protein	amtB	Nmar_1698	12
Nitrogen regulator protein PII	glnK	Nmar_1523	9
Cytochrome c oxidase	ccoI	Nmar_0184	7
Ammonia monooxygenase	amoA	Nmar_1500	14
Ammonia monooxygenase	amoB	Nmar_1500	27
Ammonia monooxygenase	amoC	Nmar_1500	8
Multicopper oxidase	mco3	Nmar_1354	5
Hypothetical protein	ompA	Nmar_1528	5
NH_4^+ transport protein	amtB	Nmar_0588	2

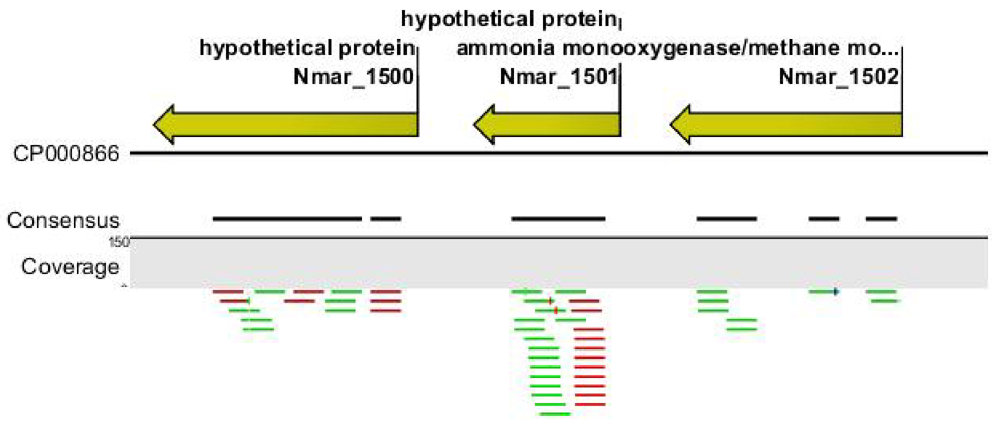

Fig. S4. Gene expression of selected *Nitrosopumilus maritimus* SCM1 genes under oxygen limitation.

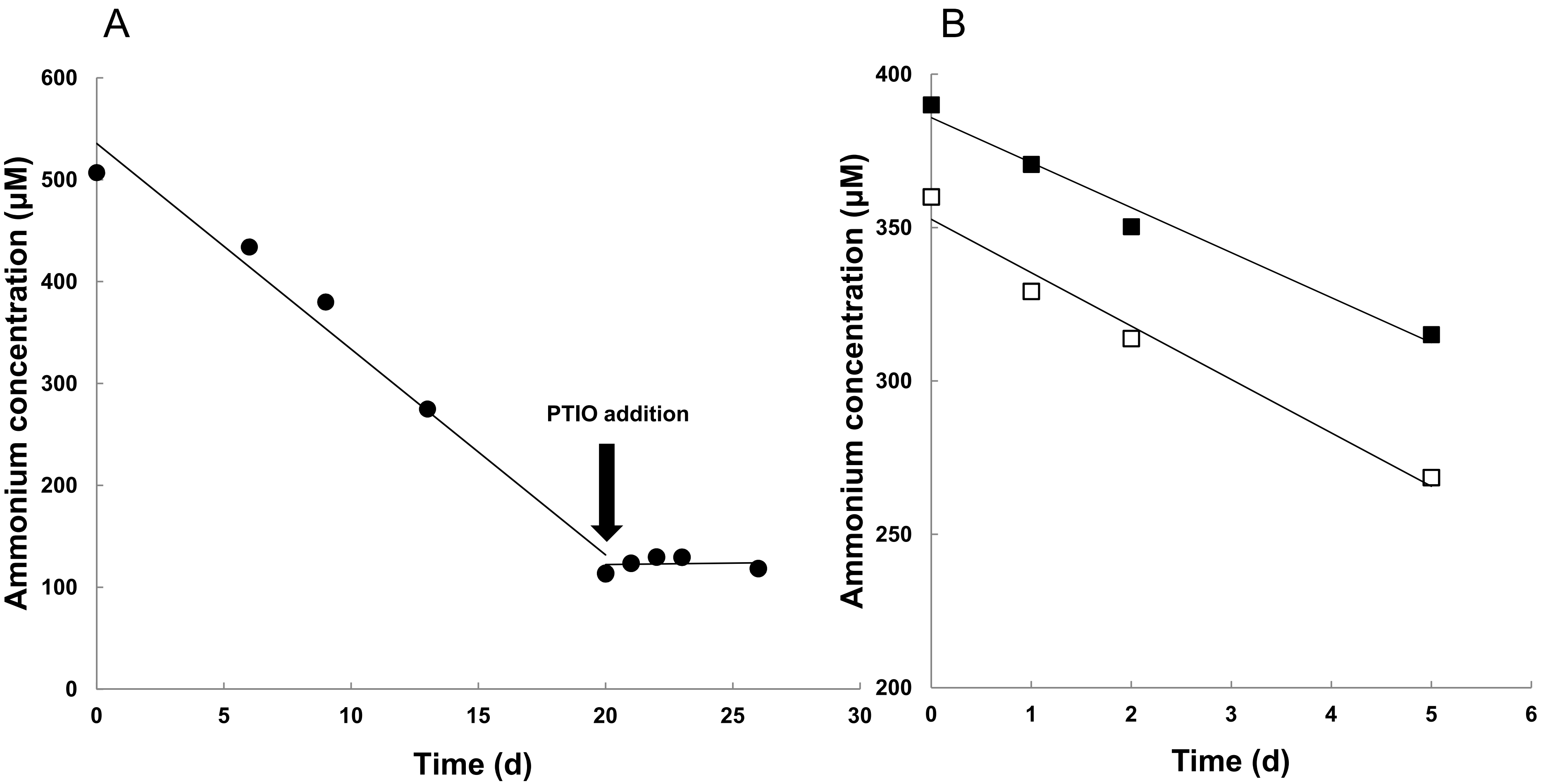

Fig. S5. Potential aerobic ammonia-oxidizing activity of the *Nitrosopumilus maritimus* culture (A) and *Nitrosomonas*-like AOB enrichment (B). Open squares (□) and closed squares (■) indicate AOB enrichment with and without PTIO addition, respectively.
