# Supplementary material for: Mimicking the oxygen minimum zones: stimulating interaction of aerobic archaeal and anaerobic bacterial ammonia oxidizers in a laboratory-scale model system
Source: Environ Microbiol. 2012 Oct 12;14(12):3146–58. doi: 10.1111/j.1462-2920.2012.02894.x (PMC3558802; doi:10.1111/j.1462-2920.2012.02894.x)
Supplement: Table S1 — Primers for PCR amplification and sequencing. [file emi0014-3146-SD2.docx]

Table S1.

| Primers for PCR amplification and sequencing | | | | |
| --- | --- | --- | --- | --- |
| Primer | Application | Sequence (5’-3’) | Specifictity | Reference |
| AmoAF | PCR/qPCR | GGGGTTTCTACTGGTGGT | amoA Bacteria | Rotthauwe et al., 1997 |
| AmoAR | PCR/qPCR | CCCCTCKGSAAAGCCTTCTTC | amoA Bacteria | Rotthauwe et al., 1997 |
| Cren AmoAF | PCR | ATGGTCTGGCTAAGACGMTGTA | amoA Thaumarchaea | Hallam et al., 2006 |
| Cren AmoAR | PCR | CCCACTTTGACCAAGCGGCCAT | amoA Thaumarchaea | Hallam et al., 2006 |
| CrenAomAQ-F | qPCR | GCARGTMGGWAARTTCTAYAA | amoA Thaumarchaea | Mincer et al., 2007 |
| CrenAomAModR | qPCR | AAGCGGCCATCCATCTGTA | amoA Thaumarchaea | Mincer et al., 2007 |
| HSF | qPCR | WTYGGKTATCARTATGTAG | hzsA anammox | This study |
| HSR | qPCR | AAABGGYGAATCATAATGGC | hzsA anammox | This study |
